# Effector and regulator: Diverse functions of *C*. *elegans* C-type lectin-like domain proteins

**DOI:** 10.1371/journal.ppat.1009454

**Published:** 2021-04-01

**Authors:** Barbara Pees, Wentao Yang, Anke Kloock, Carola Petersen, Lena Peters, Li Fan, Meike Friedrichsen, Sabrina Butze, Alejandra Zárate-Potes, Hinrich Schulenburg, Katja Dierking

**Affiliations:** 1 Department of Evolutionary Ecology and Genetics, Christian-Albrechts-Universität zu Kiel, Kiel, Germany; 2 Department of Comparative Immunobiology, Christian-Albrechts-Universität zu Kiel, Kiel, Germany; 3 Max-Planck Institute for Evolutionary Biology, Ploen, Germany; Boston Children’s Hospital, UNITED STATES

## Abstract

In *C*. *elegans*, 283 *clec* genes encode a highly diverse family of C-type lectin-like domain (CTLD) proteins. Since vertebrate CTLD proteins have characterized functions in defense responses against pathogens and since expression of *C*. *elegans clec* genes is pathogen-dependent, it is generally assumed that *clec* genes function in *C*. *elegans* immune defenses. However, little is known about the relative contribution and exact function of CLEC proteins in *C*. *elegans* immunity. Here, we focused on the *C*. *elegans clec* gene *clec-4*, whose expression is highly upregulated by pathogen infection, and its paralogs *clec-41* and *clec-42*. We found that, while mutation of *clec-4* resulted in enhanced resistance to the Gram-positive pathogen *Bacillus thuringiensis* MYBt18247 (Bt247), inactivation of *clec-41* and *clec-42* by RNAi enhanced susceptibility to Bt247. Further analyses revealed that enhanced resistance of *clec-4* mutants to Bt247 was due to an increase in feeding cessation on the pathogen and consequently a decrease in pathogen load. Moreover, *clec-4* mutants exhibited feeding deficits also on non-pathogenic bacteria that were in part reflected in the *clec-4* gene expression profile, which overlapped with gene sets affected by starvation or mutation in nutrient sensing pathways. However, loss of CLEC-4 function only mildly affected life-history traits such as fertility, indicating that *clec-4* mutants are not subjected to dietary restriction. While CLEC-4 function appears to be associated with the regulation of feeding behavior, we show that CLEC-41 and CLEC-42 proteins likely function as *bona fide* immune effector proteins that have bacterial binding and antimicrobial capacities. Together, our results exemplify functional diversification within *clec* gene paralogs.

## Author summary

C-type lectin-like domain (CTLD) containing proteins fulfill various and fundamental tasks in the human and mouse immune system. Genes encoding CTLD proteins are present in all animal genomes, in some cases in very large numbers and highly diversified. While the function of several vertebrate CTLD proteins is well characterized, experimental evidence of an immune function of most invertebrate CTLD proteins is missing, although their role in immunity is usually assumed. We here explore the immune function of three related CTLD proteins in the model nematode *Caenorhabditis elegans*. We find that they play diverse roles in *C*. *elegans* immunity, functioning as antimicrobial immune effector proteins that are important for defense against pathogen infection and probably directly interact with bacteria, but also regulators of feeding behavior that more indirectly affect *C*. *elegans* pathogen resistance. Such insight into the functional consequence of invertebrate CTLD protein diversification contributes to our understanding of the evolution of innate and invertebrate immune systems.

## Introduction

Vertebrate C-type lectin-like domain (CTLD) proteins play an essential role in pathogen recognition and subsequent activation of the immune response to fungal and bacterial infection [1]. CTLD proteins are characterized by a conserved carbohydrate-recognition domain (CRD), which may bind sugar in a calcium-dependent (C-type) manner. But not all of the proteins carrying a CRD bind glycans or require calcium for binding, which is why the more general term CTLD proteins was introduced [2]. In the vertebrate immune system CTLD proteins mainly act as antimicrobial effector proteins, classical pattern recognition receptors (PRRs), binding ligands derived from fungi, bacteria or viruses, and as dead and cancerous cell sensors enhancing tumor killing activities of natural killer cells (reviewed in [3]). In invertebrates CTLD proteins also seem to play a role in immune defenses, but their exact functions are much less clear [4].

*C*. *elegans* has a highly diversified CTLD encoding gene (*clec*) repertoire (reviewed in [4]). 288 CTLD proteins are encoded in the nematode’s genome with half of them carrying an additional CTLD or other domains such as CUB (Complement C1r/C1s Uegf Bmp1), CW (conserved cysteine and tryptophan residues), or VWA (Von Willebrand factor type A) [4, 5]. Several *C*. *elegans* transcriptome analyses revealed that the majority of *clec* genes is highly upregulated upon pathogen exposure (e.g. [6–8]) and this, in a highly specific pattern [5]. By reverse genetic analyses some *clec* genes were shown to be required for defense against infection with bacterial pathogens, e.g. *clec-17*, *clec-60*, and *clec-86* for *Microbacterium nematophilum* [9], *clec-70* for *Staphylococcus aureus* [10], *clec-65* for the pathogenic *Escherichia coli* strain LF82 [11], and *clec-174* for *Vibrio cholerae* [12]. It is however unclear, what exact role these CLEC proteins play in immune defenses. There are only four studies providing further evidence, revealing that functions of *C*. *elegans* CLEC proteins in response to pathogens are likely to be manifold: First, CLEC-39 and CLEC-49 are required for *C*. *elegans* resistance to *Serratia marcescens* infection. As both proteins bind to *S*. *marcescens in vitro*, but do not exhibit antimicrobial activity, CLEC-39 and CLE-49 were suggested to act as classical PRRs mediating pathogen recognition [13]. Second, CLEC-1 is secreted by body muscle cells and was identified as regulator of protein homeostasis in the extracellular space in response to exposure with the *B*. *thuringiensis*-derived pore forming toxin Cry5B [14]. Protein homeostasis in the extracellular space was suggested to be important for sustaining a systemic immune response by keeping secreted immune effectors functional. CLEC-1 was shown to be highly effective in preventing aggregation of LYS-7 [14], which contributes to *C*. *elegans* resistance to *B*. *thuringiensis* [6]. Third, the CTLD containing protein IRG-7 affects *C*. *elegans* pathogen resistance by activating the p38 MAPK-ATF-7 immune defense pathway [15]. Moreover, IRG-7 was identified as mediator of longevity, being a component of the reproductive longevity pathway thus revealing complex effects of a CLEC protein on *C*. *elegans* physiology [15]. Finally, the CTLD containing protein C54G4.4 was implicated in the regulation of *C*. *elegans* behavioral immune defenses. *C54G4*.*4* mutants were more resistant to pathogen infection and exhibited enhanced pathogen avoidance responses [16].

Although experimental evidence for a role of *C*. *elegans clec* genes in immune responses is scarce, *clec* genes are generally assumed to be important innate immune genes based on their pathogen-dependent expression patterns. The aim of this study was to explore to what extent pathogen-responsive *clec* genes function in *C*. *elegans* immunity. We focused on the *C*. *elegans clec* genes *clec-4*, whose expression is highly upregulated by infection with a broad array of pathogens, and its paralogs *clec-41* and *clec-42*. We explored *clec-4*, *clec-41*, and *clec-42* expression, redundancy, and their function on the gene and protein level. Moreover, we did an explorative transcriptome analysis of the *clec-4(ok2050)* mutant. We found that while *clec-41* and *clec-42* seem to encode *bona fide* immunity proteins that function in *C*. *elegans* resistance to *B*. *thuringiensis* MYBt18247 (Bt247) infection, *clec-4* indirectly affects pathogen resistance by regulating feeding behavior. Together, we demonstrate for the first time an antimicrobial function of *C*. *elegans* CLEC proteins and identified *clec-4* as novel regulator of feeding behavior.

## Materials and methods

### *C*. *elegans* strains and culture conditions

Worms were grown and maintained on nematode growth medium (NGM) agar plates seeded with *Escherichia coli* OP50 as previously described [17]. The wildtype strain N2 (Bristol) and the mutant strains RB1660 *clec-4(ok2050)* II. and HT1593 *unc-119(ed3)* III. were obtained from the Caenorhabditis Genetics Center (CGC, Minnesota, USA). The *clec-41(tm6722)* V. and *clec-42(tm6526)* V. mutant animals were obtained from the National BioResource Project (NBRP, Tokyo, Japan) [18]. Strain MY1116 *clec-4(ya1)* II. was generated using the *dpy-10* co-CRISPR strategy according to [19, 20] and subsequently outcrossed three times with the N2 strain, yielding MY1117. The double mutant MY1127 *clec-41(tm6722);clec-42(tm6526)* was obtained by crossing the respective strains. Generally, all mutant strains were outcrossed at least three time with the same wildtype N2 prior to use, *clec-4(ok2050)* mutants were outcrossed 10x, their mutations were confirmed by PCR, and the resulting lack of expression validated by RT-PCR (S2 Fig and S1A Table).

### Bacterial strains and culture conditions

*E*. *coli* OP50 was obtained from the CGC. The pathogenic *Pseudomonas aeruginosa* strain PA14 (provided by Dennis Kim) was grown first on LB plates and then in LB broth at 37°C overnight prior to inoculation of assay plates. We used *Bacillus thuringiensis* strains MYBt18247 and MYBt18679 (in the following Bt247 and Bt679; our lab strains) and Bt407 (provided by Christina Nielsen-LeRoux, INRA, France) as a non-pathogenic control. Spore-toxin mixtures were generated following previous protocols [21–23] and frozen at -20°C in aliquots with a spore concentration ranging from 3*10^9^ to 8*10^9^ particles/ml, depending on the culture, for Bt247 and 1.1*10^10^ particles/ml for Bt407. Stocks were thawed and then immediately applied in infection assays. *Serratia rubidaea* MYb239 was co-isolated with *C*. *elegans* from a compost heap in Kiel, Germany ([24], Carola Petersen and Hinrich Schulenburg). *Serratia marcescens* Db11 (provided by Jonathan Ewbank), *S*. *aureus* SA113 (provided by Andreas Peschel), and *Rhodococcus erythropolis* MYb53, which is part of the natural microbiota of *C*. *elegans* [25], were used for *in vitro* bacterial binding assays.

### Generation of transgenic *C*. *elegans* strains

The gene reporter and rescue constructs were generated by PCR fusion as previously described [26]. The promotor regions of the *clec* genes (1.0–1.6 kb upstream of start codon) were amplified from genomic DNA by PCR with primer A and primer B of which the latter contains an overlap to the sequence of the *gfp* vector (S1B Table). The *gfp* or *mCherry* coding sequence plus the 3’-UTR of *unc-54* was amplified from the Fire vector pPD95.75 with primer C (5’-agcttgcatgcctgcaggtcgact-3’) and D (5’-aagggcccgtacggccgactagtagg-3’). The transgenic constructs were finally synthesized using PCR fusion with primer A* (S1B Table) and D* (5’-ggaaacagttatgtttggtatattggg-3’) and directly injected at a concentration of 10 ng/μl. The plasmids carrying *ttx-3*p::RFP (40 ng/μl), which is expressed in the AIY interneuron pair of successfully transformed animals, or *myo-2*p::RFP (25 ng/μl), expressed in pharyngeal muscle, were used as co-injection markers in lines carrying the *clec-4* promotor construct or in lines carrying the *clec-41* or *clec-43* promotor construct, respectively. The fusion constructs with the *clec-41* and *clec-42* promotors were injected into the *unc-119(ed3)* background together with plasmid pPK605 (gift from Patricia Kuwabara, Addgene plasmid # 38148) which served as rescue for the *unc-119* phenotype (S1B Table). At least three lines were generated per reporter construct and microscopically evaluated. As the lines injected with the same fluorescent reporter construct showed similar expression patterns we focused on the ones listed in S1B Table for further analyses.

For microscopy worms were mounted on slides with a 2% agarose patch and immobilized with sodium azide. All pictures were taken with the confocal microscope LSM 700 or the Axio Observer Z.1 by Zeiss (Carl Zeiss AG, Jena, Germany).

Transgenic strains MY1121 and MY1122 for rescuing *clec-4(ya1)* were generated by injecting 50 ng/μl of a fusion construct with 0.5 kb intestinal promotor of *mtl-2* [27] and the coding region of *clec-4*, or the complete coding region of *clec-4* including 1.4 kb of the upstream promotor into the *unc-119(ed3)* background (S1C Table). The rescue for the *unc-119* mutation and *myo-2*p::RFP served as co-injection markers as described above.

### Creating *clec-4(ya1)* mutant with the CRISPR/Cas9 system

For the generation of *clec-4(ya1)* the *dpy-10* co-conversion method [19, 20] was applied. To create a deletion in the *clec-4* locus two double-stranded breaks were introduced by Cas9 and two crRNAs that correspond to the *clec-4* target sites AATCCACTAGTGCAGACTGG and GACAAGCATCTTGTTCCCGG. The repair template carrying 35 nt homology arms for *clec-4* and the *gfp* sequence was amplified from Fire vector pPD95.75 (5’-actgctcacaatcagtgaagcatcttatccaccaAGCTTGCATGCCTGCAGGTCGACT-3’ and 5’-tttgtctgtcttaaaagtgacaagcatcttgttccGGAAACAGTTATGTTTGGTATATTGGG-3’, with capital letters being the overlap to pPD95.75). The generated strain MY1116 *clec-4(ya1)*, carries a 2071 bp deletion in the *clec-4* ORF (sequence available upon request).

### RNA interference

RNAi treatment was applied to synchronized L1 larvae as previously described [28]. *E*. *coli* HT115 RNAi clones V-8P17 (in the following RNAi clone 1) and V-11P18 (in the following RNAi clone 2) from the Ahringer library were used for simultaneous knock-down of *clec-41* and *clec-42*. Both target inserts in the RNAi clones were confirmed by sequencing. The knock-down of the *clec-41* and *clec-42* genes in the RNAi-treated worms was additionally confirmed by RT-PCR (S2 Fig).

### Survival assays and lifespan analysis

PA14 requires enriched NGM (0.35% instead of 0.25% peptone) for efficient killing of worms. Agar plates seeded with a mixture of PA14 (OD_600_ 1) and *E*. *coli* OP50 (OD_600_ 5) in PBS at a concentration of 1:3 were incubated for 24 h at 37°C, followed by 24 h at 25°C. The PA14 survival assay was conducted at 25°C. For Bt infection peptone-free medium (PFM) agar plates were inoculated with a mixture of *E*. *coli* OP50 at OD_600_ 5 in PBS and Bt at different concentrations. The survival plates were kept at 20°C overnight. As different preparations of Bt247 spore-toxin mixtures may vary in pathogenicity, the exact killing levels of Bt247 may vary between experimental runs when different spore-toxin preparations are used. However, effects of knockout or knockdown of central immune system components on worm survival can still be consistently identified in comparison to simultaneously characterized controls. NGM plates for *S*. *rubidaea* infection were inoculated with an overnight culture adjusted to OD_600_ 5 in PBS and left at 20°C overnight. Plates seeded solely with *E*. *coli* OP50 or non-pathogenic Bt407 were used as controls in Bt survival assays, and *E*. *coli* OP50 plates in survival assays with PA14 and *S*. *rubidaea*. In lifespan experiments NGM plates were seeded with an overnight culture of *E*. *coli* OP50.

30 synchronized L4 larvae were picked onto each plate and worm survival was scored once after 24 h (Bt) or regularly across time until all worms on the pathogen were dead (PA14 and *S*. *rubidaea*). Worms were considered to be dead if they did not respond to light touch.

### Behavioral assays

For scoring the **avoidance behavior** on Bt 9 cm PFM plates were prepared as described above for the survival assays, but with a 30 μl spot of the bacterial mixture in the middle. For Bt exposure mild pathogen concentrations were chosen in order to challenge, but not to kill the worms. Ten synchronized L4 hermaphrodites were picked onto each bacterial spot in the middle of the plate at time point 0 hours post infection (hpi) and the worms residing on that spot were scored every second hour. The leaving index was calculated as (total number of worms—worms on bacterial spot) / total number of worms. Dead individuals were excluded from the total number of worms per plate.

For scoring the **pumping rate** on Bt PFM plates were prepared as described above for the survival assays. For Bt exposure a mild pathogen concentration was chosen. Ten synchronized L4 hermaphrodites were pipetted onto each plate to ensure that five worms are on the spot by the time the pumping rate was scored. After 6 and 24 h of exposure to Bt or OP50, the pumping of five worms per plate was counted for a period of 20 s. A total of four plates for each treatment was scored.

### Bacterial load assay

In the bacterial load assay L4 larvae were placed on PFM plates seeded with either Bt247, Bt407, or OP50 OD_600_ 5 for 24 h. All of the alive worms were picked into M9 + 0.025% Triton-X, and gravity washed with 1 ml of M9 + 0.025% Triton-X for 5 times, before worms were paralyzed with 10 mM tetramizole. Worms were then bleached with a soft bleach protocol, following [29]. Worms were then washed twice in PBS + 0.025% Triton-X, before the exact number of worms was determined (~10–25). After the final washing step, worms were homogenized in the GenoGrinder 2000 by adding sterile zirconia beads (1 mm diameter, 3 min, 3000 strokes/min). Importantly, 100 μl of washing buffer were transferred to a separate tube before grinding to be treated as the supernatant control. Worm homogenate and supernatant control were serially diluted and plated onto LB plates. After two overnights at 25°C, colonies were counted at the appropriate dilutions and colony forming units (CFUs) per worm were calculated. The soft bleaching treatment sterilized the worm surface sufficiently as the supernatant control was almost always free of viable bacteria.

### Brood size assay

For the brood size assay, single worms were picked onto NGM plates seeded with OP50, transferred daily, and the hatched offspring was scored until the end of the reproduction period. The assay was conducted at 20°C.

### *In vitro* bacterial binding assay

Proteins CLEC-4-His (50 mM Tris-HCl, 150 mM NaCl, 10% glycerol, pH 8.0), CLEC-41-His, and CLEC-42-His (both in 50 mM Tris-HCl, 150 mM NaCl, 10% glycerol, 0.5 M L-arginine, pH 8.0) were commercially obtained from GenScript (http://www.genescript.com/, Piscataway, New Jersey, USA).

The bacterial binding assay was done following published protocols [30]. In detail, four types of Gram-negative (*E*. *coli* OP50, *P*. *aeruginosa* PA14, *S*. *marcescens* Db11, *S*. *rubidaea* MYb239) and 4 types of Gram-positive bacteria (*S*. *aureus* SA113, *B*. *thuringensis* Bt247 *and* MYBt18679, *and R*. *erythropolis* MYb53) were grown at 37°C or 28°C overnight in LB to mid-logarithmic phase, pelleted, washed, and resuspended in TBS buffer with CaCl_2_ (50 mM Tris, 150 mM NaCl, 2 mM CaCl_2_, pH 7.5) at OD_600_ 2. 100 μl bacterial solution was incubated with 6 μg recombinant protein at gentle rotation for 1 h at room temperature. The bacteria were washed three times with 1 ml TBS-CaCl_2_ and eluted with 100 μl of 2% SDS. The whole lysates plus 5x loading buffer were heated at 95°C for 5 min, equally loaded onto a 12% SDS-PAGE, analyzed by Coomassie staining, and then transferred to a PVDF transfer membranes (BIO-RAD, Cat. #1704272). After blocking in 5% non-fat milk at room temperature for 1 h the membranes were incubated with Mouse-anti-His mAb (BIO-RAD, Cat. #MCA1396GA) overnight at 4°C, followed by HRP-linked secondary antibody (advansta, Cat. #R-05071-500). The signals were detected using the chemiluminescence phototype-HRP kit (BIO-RAD, Cat. #1705060S) according to the manufacturer’s instructions.

### Antimicrobial activity assay

Assessing the antimicrobial activity of CLEC proteins *in vitro* was adapted from a previously published broth dilution method (Protocol (E), [31]).

CLEC proteins were serially diluted in LB in a polypropylene 96-well plate leaving 50 μl protein dilution per well. An *E*. *coli* OP50 culture in logarithmic phase was diluted in LB to a final concentration of 100–1,000 CFUs/well, 50 μl were added to each well, and the plates were incubated at 37°C overnight. The control wells contained either only serial dilutions of the CLEC’s native buffer and OP50 (buffer control), only LB (sterility control), or OP50 in LB (growth control). Melittin (Sigma-Alrich, Cat. M2272), an antimicrobial peptide of the honeybee venom, served as positive control. The minimal inhibitory concentration (MIC) was defined as the lowest protein concentration that inhibited visibly bacterial growth, i.e. no bacterial pellet or a diffuse bacterial pellet without defined border at the bottom of the well. The wells with the resulting MICs were plated onto LB plates and incubated overnight in order to determine the CLEC’s bactericidal or bacteriostatic activity.

For testing the synergistic effect of two CLEC proteins one CLEC protein was mixed with either the second CLEC protein or with the native CLEC buffer in a polypropylene 96-well plate and serially diluted in the CLEC buffer, leaving 50 μl protein dilution per well. A Bt407 or Bt247 culture in logarithmic phase was diluted in LB to a final concentration of approx. 2,000 CFUs/well, 50 μl were added to each well, and the plates were incubated at 28°C overnight.

### Meta-analysis of pathogen-dependent expression of *clec-4* and its paralogs

Information on *clec-4* and its paralogs were downloaded from WormBase Version WS250 (WormBase web site, http://www.wormbase.org, release WS250) [32], gene expression data sets from the category “Microbes” (pathogens) were analyzed using WormExp (http://wormexp.zoologie.uni-kiel.de/wormexp/)) [33]. Only the data sets, in which *clec-4* was differently expressed are shown.

### *clec-4(ok2050)* transcriptome analysis by RNA-Seq

Transcriptomic responses were assessed 6 and 12 h after exposure to the respective bacteria. At the respective time points, worms were washed off the assay plates with PBS containing 0.3% Tween20, and subsequently centrifuged. The worm pellet was resuspended in 800 μl TRIzol (Life Technologies) reagent and worms were broken up prior to RNA extraction by treating the worm suspension five times with a freeze-and-thaw cycle using liquid nitrogen and a thermo block at 45°C. RNA was extracted using a NucleoSpin miRNA extraction kit (Macherey-Nagel), treated with DNAse, and stored at -80°C. RNA libraries were prepared for sequencing using standard Illumina protocols. Libraries were sequenced on an Illumina HiSeq 2000 sequencing machine with paired-end strategy at read length of 100 nucleotides. The raw data is available from the GEO database [34, 35] under GSE110913.

RNA-Seq reads were firstly trimmed for adaptor sequence, masked for low quality sequence via Trimmomatic [36] and then mapped to the *C*. *elegans* genome (WormBase web site, http://www.wormbase.org, release WS235) by STAR 2.5.3a [37] under default setting. Transcription abundance (read counts per gene) was extracted via HTSeq [38]. Differential expression analysis was performed by aFold from ABSSeq [39]. We only considered genes with a significant change between conditions (*clec-4* mutant vs. N2; adjusted *p*-value < 0.01). The log_2_ transformed fold-changes were taken as input for k-means cluster analysis using cluster 3.0 [40]. A heatmap was generated by TreeView version 1.1.4r3 [41].

### Gene ontology and gene set enrichment analysis

Gene ontology (GO) analysis was performed using DAVID with a cutoff of FDR < 0.05 [42]. Gene set enrichment analysis was performed by WormExp [33]. A gene set with FDR < 0.05 was considered significant.

### Statistical analyses

Statistical analyses were done with RStudio (Version 1.0.136), graphs created with its package ggplot2 (Version 2.2.1) and edited with Inkscape (Version 0.91). General statistical tests and appropriate corrections for multiple testing were applied and can be found in S2 Table.

## Results & discussion

### Expression of *clec-4* and its paralog *clec-41* is highly upregulated upon exposure to various pathogenic bacteria

**Fig 1:**
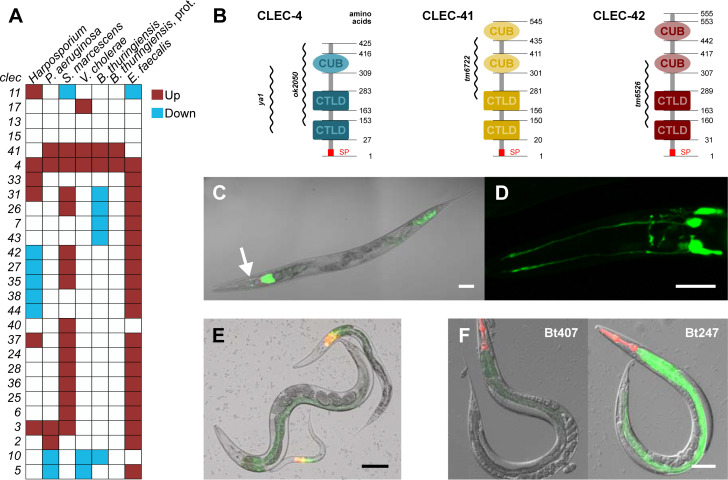
*clec-4*, *clec-41*, and *clec-42* expression and protein domain architecture. (A) Heatmap showing the differential expression of *clec-4* and a subset of its paralogs in worms exposed to different pathogens. Note that one Bt column is based on proteome data (prot.). 15 *clec-4* paralogs, which did not exhibit a differential expression have been excluded. Genes are vertically sorted by hierarchical clustering using Cluster 3.0 [40]. Red and blue colors indicate up- and downregulation, respectively. Transcriptomic data taken from previously published studies [7, 12, 45, 47–49] and GSE110913 were analyzed using WormExp [49]. (B) Domain architecture of the CLEC-4, CLEC-41, and CLEC-42 proteins adapted from SMART (http://smart.embl-heidelberg.de/) taking UniProt as source database. Numbers represent the amino acid position, the regions affected by deletions in the respective mutants are indicated on the side. SP = signal peptide. (C-F) *In vivo* expression of *clec-4*, *clec-41*, and *clec-42*. (C, D) Expression of *clec-4*p::GFP (C) throughout the intestine (most strongly in the first intestinal ring (int1) and the posterior intestine), in the amphid neurons (white arrow) of a L4 larva, and (D) in the amphid neurons and amphid nerves in the head of a L1 larva. The scale bar represents 50 μm in (C) and 10 μm in (D). (E) Simultaneous expression of *clec-41*p::GFP and *clec-42*p::mCherry throughout the intestine and in int1 at different larval stages, respectively. The scale bar represents 100 μm. (F) Expression of *clec-41*p::GFP in the intestine upon exposure to the non-pathogenic Bt407 and the pathogenic Bt247. The co-injection marker *myo-2*p::mCherry is expressed in the pharynx. The scale bar represents 100 μm. Also see S1 Fig.

*C*. *elegans clec* genes have repeatedly been suggested to be involved in pathogen defense because they are always among the genes that are highly upregulated by pathogen infection. The repertoire of induced *clec* genes differs from pathogen to pathogen, suggesting a highly specific regulation (reviewed in [4]). However, the expression of a few *clec* genes is activated by infection with several different pathogens, indicating a more general role of these genes in *C*. *elegans* defense responses. We performed a transcriptome meta-analysis of pathogen-dependent *clec* gene expression using WormExp [33] and identified *clec-4* as one of the *clec* genes, which is highly upregulated upon infection with a broad array of pathogens, including Gram-negative and Gram-positive bacteria, as well as the fungal intestinal pathogen *Harposporium spec*. (Fig 1A). The pathogen-dependent upregulation of *clec-4* was previously confirmed for the Gram-negative *P*. *aeruginosa* strain PA14 by qRT-PCR [43] and on the protein level for the Gram-positive *B*. *thuringiensis* strain Bt247 by a quantitative proteome analysis [44, 45].

Evolution of *C*. *elegans clec* genes is likely subjected to repeated duplication events [5]. *clec-4* has 39 paralogs according to WormBase (http://www.wormbase.org, release WS250) which might function redundantly [46]. Since co-expressed genes are predicted to be involved in the same cellular process, we examined the pathogen-induced expression of these 39 *clec-4* paralogs to identify co-expressed genes. The expression of the gene *clec-41* was upregulated like the expression of *clec-4* in five out of seven pathogen data sets (Fig 1A), in case of Bt infection at both the transcriptome and proteome level [44, 45]. In addition to *clec-41*, we decided to include *clec-42*, the closest paralog to *clec-41*, in our analysis. *clec-42* is the only other gene among the 39 paralogs that encodes a protein with the same domain architecture as CLEC-41, consisting of two CTL and two CUB domains (Fig 1B). We therefore focused our functional analysis on these three genes.

### *clec-4*, *clec-41*, *and clec-42* are expressed in the intestine and co-expressed in int1

To elucidate *in vivo* expression patterns of *clec-4*, *clec-41*, and *clec-42* we generated transgenic strains carrying the transcriptional reporter construct *clec-4*p::GFP (Fig 1C and 1D) and strains carrying the *clec-41*p::GFP reporter alone (Fig 1F) or together with *clec-42*p::mCherry (Fig 1E). Under standard laboratory culture conditions, *clec-4*p::GFP was constitutively expressed in the intestine of worms throughout all developmental stages (S1A Fig). However, expression of *clec-4*p::GFP seemed to be decreased in L4s and adults, in which a strong GFP signal could only be observed in the first intestinal ring (int1) and the posterior intestine (Fig 1C). Localized intestinal expression of infection-induced genes was observed previously: For example, expression of the caenopore gene *spp-7* is stronger in the posterior intestine than in the anterior [50]. Mallo et al reported expression of the lysozyme gene *lys-1* throughout the intestine, but also observed *lys-1*::GFP in vesicles in a single posterior intestinal cell [51]. In addition to its intestinal expression, *clec-4*p::GFP is constitutively expressed in amphid neurons from the first larval to the adult stage (Fig 1C and 1D). Expression of the *clec-4*p::GFP reporter gene was not inducible after an infection with pathogenic Bt or PA14, which is in contrast to the strong upregulation shown in the gene expression analyses. It was, however, consistently observed across experiments and pathogens (Fig 1A), including both transcriptomic and also independently performed proteomic analyses on the same Bt247 pathogen [44, 45, 52] (Fig 1A). This discrepancy indicates that the extrachromosomal array does not faithfully recapitulate endogenous expression of *clec-4*. One possible reason might be that the reporter gene construct does not contain all relevant regulatory sequences.

Similar to *clec-4*, *clec-41*p::GFP is weakly but constitutively expressed throughout the intestine of worms at all life stages under standard laboratory culture conditions. In contrast, *clec-42*p::mCherry is constitutively expressed only in int1 and only at the four larval stages, not in adults (Fig 1E). Interestingly, a transcriptional GFP reporter of another *clec-4* paralog, *clec-43*, was also exclusively expressed in int1 (S1B Fig). That some *clec* genes are specifically expressed in int1 is an intriguing observation. The intestine of the worm is a tube consisting of 20 epithelial cells, of which the most anterior ring (int1) directly behind the pharynx is comprised of four cells, whereas the subsequent rings (int2—int9) are comprised of two cells each (https://wormatlas.org/). It was already noted by John Sulston and colleagues that “The anterior ring of four cells (int1) is specialized in having shorter microvilli than the rest of the intestine” [53]. Moreover, the pH in the lumen of int1 differs from the pH in the remaining intestine and the int1-associated part of the gut was suggested to act as mediator between the basic pharynx and the acidic intestine [54]. Our striking observation of exclusive expression of several *clec* genes in int1 provide further evidence of a specialization of int1. The secretion of CLEC proteins and other potential immune effectors specifically by int1 might create a distinct microenvironment that is important for host-microbe interactions at the ‘entry gate’ of the intestine.

Although *clec-41*p::GFP is already constitutively expressed, we observed an even stronger expression after infection with Bt247 (Fig 1F), which confirms the infection-dependent upregulation seen in the expression data sets. The expression of *clec-42*p::mCherry was not induced by Bt247 infection, which also is in line with the expression data. Together, *clec-4* and *clec-41* are both expressed in the *C*. *elegans* intestine and their expression co-localizes with *clec-42* expression in the anterior intestinal ring (int1).

### *clec-4* mutants exhibit enhanced survival on *B*. *thuringiensis*

**Fig 2:**
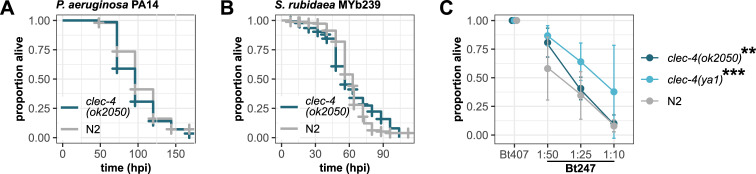
*clec-4(ok2050)* and *clec-4(ya1)* increase *C*. *elegans* resistance to Bt247 infection. Survival of *clec-4(ok2050)* and N2 wildtype worms on (A) *P*. *aeruginosa* PA14 and (B) *S*. *rubidaea* MYb239 over time. (A, B) Alive, dead, and missing worms were scored until all individuals on the pathogen were dead. No significant differences in survival between *clec-4* mutants and N2 wildtype worms as determined by Kaplan-Meier analysis [55] and log-rank test [56]. Horizontal ticks represent censored data (missing worms), n = 5, (A) data are representative of two independent experiments, (B) data represent the mean of three independent experiments. (C) Difference in survival on serial dilutions of Bt247 and a dilution of 1:10 of the non-pathogenic Bt407 control 24 hpi (hours post infection) between *clec-4(ok2050)*, *clec-4(ya1)*, and N2. Means ± standard deviation (SD) of n = 4 are shown, data are representative of at least four independent experiments. **p < 0.01, and ***p < 0.001, according to a generalized linear model (GLM) [57]. Also see S3A and S3B Fig, and S2A and S3A–S3C Tables.

*clec-4* expression is highly upregulated in response to infection with several different pathogens, including *P*. *aeruginosa* PA14, *S*. *marcescens* Db10, and *B*. *thuringiensis* Bt247 (Fig 1A). As a first step toward understanding the potential functional role of *clec-4* in *C*. *elegans* immune defense responses, we tested if *clec-4* is required for resistance to infection with the Gram-negative pathogens PA14 and *S*. *rubidaea* MYb239, and the Gram-positive pathogen Bt247, using the *clec-4(ok2050)* deletion mutant. The *clec-4(ok2050)* mutant contains a deletion of 1610 bp, which removes most of the coding region (S2G Fig), comprising part of one CTLD at the N-terminus and the complete remaining CTLD and CUB domain, resulting in the complete absence of any mRNA product and thus represents a null deletion allele (Figs 1B and S2A). We found that *clec-4(ok2050)* mutant animals survived as well as wildtype worms on PA14 and *S*. *rubidaea* (Fig 2A and 2B), being consistent with previous results on the unaffected susceptibility of the *clec-4(ok2050)* mutant to infection with *S*. *marcescens* [13]. These results indicate that *clec-4* does not function in *C*. *elegans* defense responses to these pathogens. On the Gram-positive pathogens Bt247 however, *clec-4(ok2050)* mutant animals were unexpectedly more resistant than wildtype worms (Fig 2C).

To confirm that the knock-out of *clec-4* is the causative factor of the resistance phenotype we used CRISPR/Cas9 to generate the *clec-4(ya1)* allele that contains a large in-frame deletion, which removes a 2071 bp long fragment of the *clec-4* ORF (S2G Fig), i.e. the complete intermediate CTLD, the majority of the N-terminal CTLD, and part of the CUB domain, resulting in a very short mRNA product (Figs 1B and S2B). *clec-4(ya1)* animals were also more resistant to Bt247 infection (Fig 2C). It is important to note that the survival phenotypes of both *clec-4(ok2050)* and *clec-4(ya1)* mutant animals were variable across several experimental runs (S3A and S3B Fig). Taking all data into account, the resistance phenotype was, however, the most prevalent *clec-4* mutant phenotype, observed in 7 out of 12 experimental runs for *clec-4(ok2050)* and 3 out of 4 runs for *clec-4(ya1)*. Also, we generated two strains, in which we reintroduced copies of the *clec-4* sequence expressed from its endogenous promoter and from an intestinal promoter, respectively, into the *clec-4(ya1)* knock-out mutant background and obtained partial rescue of the resistance phenotype observed in *clec-4(ya1)* mutant animals (S3C Fig). As this particular Bt247 infection assay produces highly reproducible results for gene mutants with a central role in resistance (see e.g., our previous results with the assay for RNAi-silenced *elt-2* or *jun-1* knock-out mutants [52]), we conclude that mutations in *clec-4* contribute partially albeit not essentially to *C*. *elegans* resistance to Bt247 infection.

### Functional loss of CLEC-4 increases feeding cessation on pathogenic Bt247 and consequently decreases intestinal pathogen load

**Fig 3:**
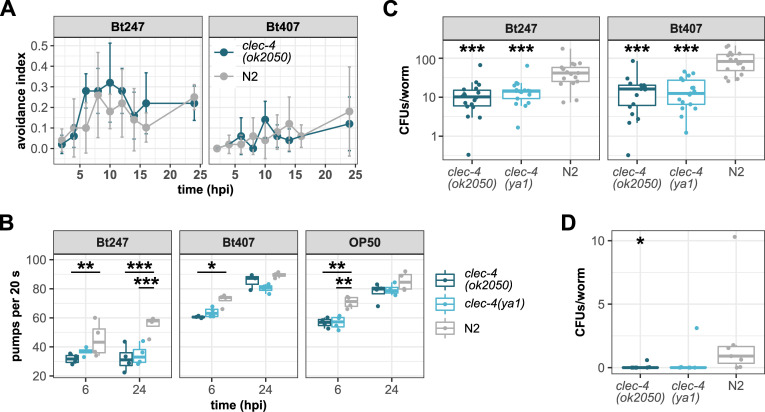
*clec-4(ok2050)* and *clec-4(ya1)* decrease *C*. *elegans* feeding leading to a reduced bacterial load. (A) Avoidance behavior of *clec-4(ok2050)* and N2 wildtype worms on Bt247. Worms were exposed to a mild concentration (1:400) of non-pathogenic Bt407 and pathogenic Bt247. The avoidance index is defined as (initial total number of worms–worms on bacterial spot) / initial total number of worms. Means ± SD of n = 5, no significant differences between *clec-4(ok2050)* and N2 were determined by a generalized linear mixed model (GLMM) [59] per bacterial treatment, *p*-values corrected for multiple comparisons with Bonferroni [60]. (B) Pumping rate of *clec-4(ok2050)*, *clec-4(ya1)*, and N2 wildtype worms on a 1:100 dilution of Bt247 and Bt407, n = 4, data are representative of three independent experiments. *p < 0.05, **p < 0.01, ***p < 0.001, according to GLMM per bacterial treatment and per worm strain, Bonferroni corrected [60]. (C, D) Bacterial load on (C) a Bt dilution of 1:100 or (D) OP50 measured in colony forming units (CFUs) per worm. Shown are pooled data, (C) n = 18 or (D) n = 16 combined from three independent experiments. *p < 0.05, ***p < 0.001, as determined by Wilcoxon rank sum test, Bonferroni corrected [60]. Also see S2B and S3D–S3F Tables.

Despite the upregulation of *clec-4* gene expression [44] and the higher abundance of CLEC-4 protein [45] upon Bt247 infection, *clec-4* deficiency unexpectedly led to increased resistance (Fig 2C). It is possible that pathogen resistance results from defense behaviors such as increased pathogen avoidance or decreased pharyngeal pumping (i.e. pathogen up-take) as previously shown for the CTLD containing gene *C54G4*.*4* mutant [16]. Also, we observed *clec-4* expression in *C*. *elegans* amphid neurons (Fig 1D), which are chemosensory and thermosensory neurons with openings to the exterior that play a role in detecting microbial cues and in regulating pathogen avoidance behavior [58]. To understand if *clec-4* functions in behavioral defense, we assessed avoidance and pumping activity of the *clec-4* mutants on Bt247. We found that *clec-4* mutant animals avoided the pathogenic Bt247 strain as much as wildtype worms (Fig 3A). However, *clec-4* mutants exhibited a prolonged decrease in pharyngeal pumping on Bt247: As expected N2 wildtype animals decreased their pumping activity on pathogenic Bt247 compared to the non-pathogenic control 6 hpi. Pumping activity of *clec-4* mutants was even significantly lower than that of wildtype animals and while the wildtype N2 strain resumed feeding at 24 hpi, the *clec-4* mutants remained at a low feeding rate (Fig 3B).

We then examined whether *clec-4* deficiency affects pathogen accumulation in the intestine. To this end, we quantified intestinal pathogen load by counting colony forming units (CFUs) of live bacterial cells recovered from the intestines of wildtype and mutant animals. To avoid any contamination with bacterial cells that stick to the surface of the worm, we applied a mild bleaching protocol that efficiently removed all bacteria from the outside (see [29] and materials and methods). We observed a substantial reduction in pathogen load in *clec-4(ok2050)* and *clec-4(ya1)* animals compared to wildtype worms (Fig 3C).

These results indicate that the *clec-4* mutant animals’ enhanced survival on Bt247 is due to a reduction in pumping and consequently a reduction in pathogen load. As feeding cessation on pathogens is a behavioral response and as behavior of animals can be dramatically affected by small changes or variations in culture or assay conditions [61], this may explain the variation we observed in *clec(ok2050)* and *clec(ya1)* resistance to Bt247 (S3A and S3B Fig). *clec-4* mutants were as resistant as wildtype worms to infection with *S*. *rubidaea* MYb239 (Fig 2B) and PA14 (Fig 2A). Interestingly, *C*. *elegans* does not exhibit feeding cessation on all pathogenic bacteria: *C*. *elegans* feeds normally on the pathogens *P*. *aeruginosa* PA01 and *Salmonella Typhimurium* MST1 [62]. On PA14, worms exhibit pathogen avoidance behavior, but do not decrease pharyngeal pumping [63]. Moreover, *eat-1* mutants, which also exhibit a reduced rate of pharyngeal pumping [64] are indistinguishable from wildtype for resistance to PA14 [63] and pharyngeal pumping does not affect PA14 accumulation in the intestine [65]. Consequently, killing by PA14 appears to depend on the establishment and proliferation of bacteria within the gut and to be independent of the rate at which bacteria enter the gut [63]. Thus, prolonged feeding cessation as exhibited by the *clec-4* mutants only leads to an increase in resistance to infection when the rate of pathogen intake determines the rate of pathogen accumulation in the gut, as is the case for Bt.

If *clec-4* regulates feeding behavior, why is its expression upregulated during pathogen infection (Fig 1A)? Most transcriptome analyses of *C*. *elegans* pathogen responses determine pathogen-induced genes through a comparison with the expression pattern shown on *E*. *coli* OP50. However, in contrast to *E*. *coli*, pathogenic bacteria colonize the intestine of young worms and live bacteria accumulate in the gut. We thus hypothesized that *clec-4* expression is not only induced by pathogens, but by live, colonizing bacteria in general. Members of the beneficial natural *C*. *elegans* microbiota are known to colonize the *C*. *elegans* intestine and we thus looked for *clec-4* expression in two transcriptome data sets on the *C*. *elegans* response to its natural microbiota. *clec-4* expression is indeed also upregulated on these non-pathogenic bacteria [66]. This observation points to a modulation of *clec-4* expression depending on the quantity or nutritional composition of bacterial food in the gut and is in agreement with a role of *clec-4* in regulating feeding also on non-pathogenic bacteria (see below).

### Functional loss of CLEC-4 affects feeding

During the pumping assays we observed that *clec-4* depletion also led to a pumping/feeding phenotype on Bt407 that we used as non-pathogenic control and on the food bacterium *E*. *coli* OP50 (Fig 3B). However, the pumping phenotype was more subtle and we detected a statistically significant difference in pumping between wildtype and *clec-4* mutants in two out of three runs at only one of two time points on Bt407 and in one of three runs on *E*. *coli* (S2B Table). We then measured bacterial load by counting CFUs and found that bacterial load was significantly reduced in *clec-4* mutants for both Bt407 (Fig 3C) and *E*. *coli* (Fig 3D). However, as *E*. *coli* OP50 bacteria are efficiently broken up by the grinder in the *C*. *elegans* pharynx [64] and live *E*. *coli* cells thus do usually not accumulate in the gut of young worms (day 1 adults in our experiments), the CFU counts were very low (ranging from 0 to 19 for *clec-4* mutant animals and from 0 to 75 for wildtype animals). Also, *clec-4* mutants showed normal development and morphology. It is thus difficult to draw any conclusions on differences in the nutritional status of the animals from these experiments.

### Gene expression profile links *clec-4* to nutrient sensing

**Fig 4:**
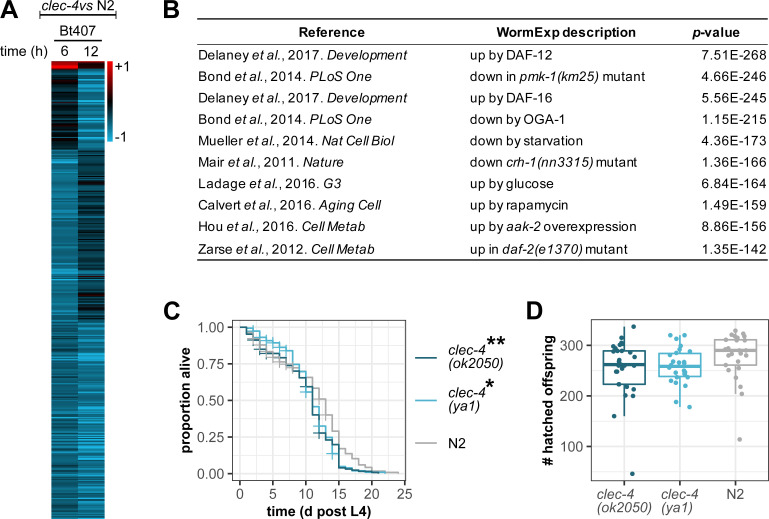
Transcriptional and WormExp EASE analysis and life history traits of *clec-4(ok2050)* and *clec-4(ya1)*. (A) Heatmap representing all significant differentially expressed (DE) genes 6 h and 12 h after exposure to non-pathogenic Bt407, comparing *clec-4(ok2050)* with wildtype N2. (B) Overview of enrichment of WormExp gene sets [70–77], inferred from Expression Analysis Systematic Explorer (EASE) analysis on differentially expressed genes 6 h and 12 h after exposure to non-pathogenic Bt407, comparing *clec-4(ok2050)* with wildtype N2. *p*-values are Benjamini-Hochberg corrected. (Also see S5A and S5B Table). (C) Lifespan analysis of *clec-4(ok2050)* and *clec-4(ya1)* mutants and N2 wildtype animals under standard conditions. Lifespan analyses led to inconclusive results as lifespan curves of *clec-4* mutants crossed the curve of N2, n = 5, data are representative of three independent experiments (also see S4A and S4B Fig). *p < 0.05, ***p < 0.001, according to Kaplan-Meier analysis [55] and log-rank test [56]. Horizontal ticks represent censored data (missing worms). (D) Lifetime brood size of *clec-4(ok2050)* and *clec-4(ya1)* mutants and N2 wildtype animals. Shown are pooled data, n = 30 combined from three independent experiments. No significant differences between worm strains as determined by Wilcoxon rank sum test. Also see S4C and S4D Fig, and S2C, S3G and S3H Tables.

To explore the possibility that the restrictions in normal feeding observed for the *clec-4* mutants result in a transcriptional response similar to the response to dietary restriction or starvation, we performed gene expression profiling of the *clec-4(ok2050)* mutant and wildtype animals exposed to *E*. *coli* OP50 and non-pathogenic Bt407. We confirmed that the Bt407 and *E*. *coli* OP50 treatment showed only little variation to each other [52], validating that Bt407 is non-pathogenic. 435 genes were differentially regulated in *clec-4(ok2050)* mutant worms on Bt407 when compared to wildtype worms, all, except 11 of them, downregulated (Fig 4A, S5A Table). We used WormExp [49] for an enrichment analysis to assess the overlap between the *clec-4*-dependent gene set and other, previously published *C*. *elegans* gene sets and found that one common denominator within the most significantly enriched gene sets was nutrient sensing. For example, targets of the p38 MAPK pathway (“down in *pmk-1 mutant”*) and insulin/insulin-like growth factor-1 (IGF-1) signaling (“up by DAF-16”, “up in *daf-2* mutant”) were enriched in the genes downregulated in *clec-4* mutants (Fig 4B and S5B Table). The p38 MAPK pathway is a crucial *C*. *elegans* innate immunity pathway [67] and has more recently been proposed to act as an immunometabolic pathway that senses bacterial and nutrient signals [68]. *daf-2*, which encodes the *C*. *elegans* insulin/IGF-1 receptor ortholog, and *oga-1*, which encodes a O-GlcNAc cycling enzyme that is a component of carbohydrate metabolism/hexosamine signaling, play central roles in nutrient sensing [69]. Furthermore, genes regulated by starvation, glucose, rapamycin, the dietary and environmental sensor DAF-12, the *C*. *elegans* AMP/energy signaling AMP-activated protein kinase alpha subunit AAK-2, and the homolog of the cyclic AMP-response element binding protein (CREB) CRH-1, were enriched in genes downregulated in *clec-4* mutants. While the CREB gene *crh-1* has not as yet been directly linked to dietary restriction and/or starvation, activation of the energy-sensing AMPK AAK-2 and inhibition of the nutrient-sensing target of rapamycin (TOR) pathway by rapamycin are perturbations mimicking dietary restriction. Together, the results of the enrichment analysis indicate an involvement of *clec-4* in energy metabolism and/or nutrient sensing.

### CLEC-4 loss of function has subtle effects on reproduction

Dietary restriction affects life history traits in many organisms, including *C*. *elegans* [78]. The *C*. *elegans* dietary restriction mutant *eat-2* has a reduced food intake due to a mutational defect in pharyngeal pumping [64, 79]. *eat-2* mutation has been shown to increase lifespan [79], extend the self-fertile reproductive period [80, 81], and reduce lifetime fertility [82]. To assess if the restrictions in food intake similarly affect life history traits in *clec-4* mutants, we measured lifespan, fertility, and reproductive timing in *clec-4(ok2050)* and *clec-4(ya1)* mutants. The data on lifespan were inconclusive, as the curves of the *clec-4* mutants crossed that of wildtype worms in two experimental runs and showed no difference in another experimental run (Figs 4C, S4A and S4B). However, the brood size of *clec-4* mutants was smaller than that of wildtype worms (Fig 4D), albeit the difference was statistically significant only during early reproduction (S4C Fig) and there was no statistically significant difference in overall lifetime brood size (Fig 4D). In comparison to wildtype worms, early progeny production was slightly decreased and late reproduction was increased in *clec-4* mutants, but there was no extension of the reproductive period (S4D Fig). Thus, mutations in *clec-4* do only have an effect on fertility during early reproduction, but do not subject worms to dietary restriction as mutations in *eat-2*. In this context it is important to note that the magnitude of the effect of mutations in *eat-2* on life history traits such as lifespan are correlated with the severity of the eating defect [64, 79] and that longevity induced by mutation in *eat-2* has been shown to be variable [83]. As *clec-4* mutants only have a milder pharyngeal pumping phenotype, the defect in normal feeding behavior may alter the nutritional state of the animals that is reflected in their gene expression profile (Fig 4A and 4B), but food intake appears to be sufficient to support normal lifespan and fertility.

*C*. *elegans* feeding behavior is influenced by the presence and quality of bacterial food and by internal nutrient status [84, 85]. How nutrient status is sensed and transduced is not well understood. CLEC-4 is predicted to have a signal sequence (as 81% of all *C*. *elegans* CLEC proteins) and may thus be secreted into the intestinal lumen, where it may bind bacterial compounds or internal nutrients such as glucose or other glycans. Only one *C*. *elegans* CLEC protein was found to have sugar-binding activity so far: CLEC-79 binds to the non-reducing terminal galactose residues of glycans [86]. We thus aimed at further investigating CLEC-4 function also on the protein level and first analyzed binding of a recombinant CLEC-4 protein (production of the protein was outsourced to a specialized company; see materials and methods) to Gram-positive and Gram-negative bacteria. Recombinant CLEC-4 did not bind to any of the tested bacteria (see below). Also, we conducted a natural *C*. *elegans* N-glycan microarray [87] to investigate CLEC-4-carbohydrate interactions, but CLEC-4 did not bind to any carbohydrates included in the glycan array. It is difficult to interpret these negative results. CLEC-4 may still be a carbohydrate-binding protein, targeting motifs on other glycoconjugates (e.g., O-glycan and glycolipids) that were not included in the N-glycan array. We also cannot exclude that the recombinant protein is inactive due to misfolding, the lack of post-translational modifications, which may be required for its proper function, in the *E*. *coli* expression system, or missing co-factors. Thus, we were unable to determine the role, if any, of CLEC-4 in binding bacteria or glycans.

### Simultaneous knock-down of *clec-41* and *clec-42* increases susceptibility to Bt247

**Fig 5:**
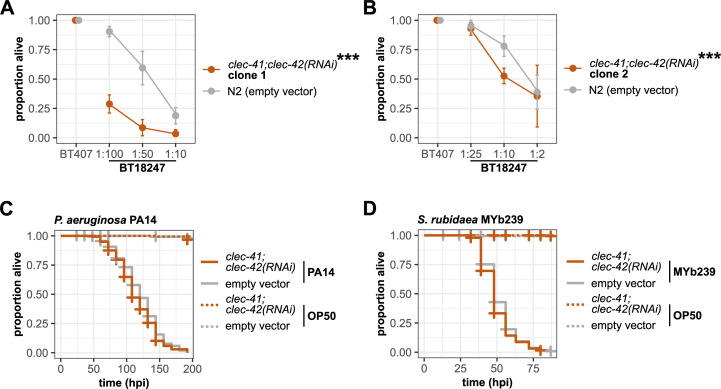
Simultaneous knock-down of *clec-41* and *clec-42* increases susceptibility to Bt247. (A, B) Difference in survival on serial dilutions of Bt247 and a dilution of 1:10 of the non-pathogenic Bt407 control 24 hpi between *clec-41;clec-42(RNAi)* worms and empty vector control worms using either (A) RNAi clone 1 (Ahringer library V-8P17) or (B) RNAi clone 2 (Ahringer library V-11P18). Means ± standard deviation (SD) of n = 5 are shown. Data are representative of five independent experiments. ***p < 0.001, according to a generalized linear model (GLM) [57]. (C, D) Survival of *clec-41;clec-42(RNAi* clone 1*)* and RNAi empty vector control worms on (C) *P*. *aeruginosa* PA14 and (D) *S*. *rubidaea* MYb239 over time. Alive, dead, and missing worms were scored until all individuals on the pathogen were dead. No significant differences in survival between *clec-41;clec-42(RNAi)* and empty vector control worms as determined by Kaplan-Meier analysis [55] and log-rank test [56]. Horizontal ticks represent censored data (missing worms), n = 5. Also see S5A and S5B Fig, and S2D, S3I and S3J Tables.

The meta-analysis of pathogen-dependent expression of *clec-4* and its paralogs revealed that *clec-41* is co-expressed with *clec-4* (Fig 1A) As co-expression might indicate similar function, we explored function of *clec-41* and its closest paralog *clec-42*. First, we used two RNAi clones from the Ahringer library, which both simultaneously target *clec-41* and *clec-42* and reduce mRNA levels of both genes (S2F Fig). In contrast to *clec-4* deficiency, silencing *clec-41;clec-42* expression in wildtype worms caused increased susceptibility to Bt247 infection (Figs 5A, 5B, S5A and S5B), but did not affect susceptibility to infection with PA14 or *S*. *rubidaea* MYb239 (Fig 5C and 5D). This indicates that *clec-4* and *clec-41*/*clec-42* have distinct functions and that certain *C*. *elegans clec* genes indeed function in defense against specific pathogens, as previously suggested [4].

To disentangle the roles of *clec-41* and *clec-42* in mediating resistance to Bt247 and to confirm the results of the RNAi experiments, we assessed survival of *clec-41(tm6722)* and *clec-42(tm6526)* single mutants and a *clec-41(tm6722)*;*clec-42(tm6526)* double mutant, respectively. However, the results were inconclusive. While the *clec-41* single mutant was as resistant as wildtype worms (S5C Fig), *clec-42(tm6526)* animals were significantly more resistant than wildtype worms in two runs and more susceptible in three experimental runs (S5D Fig). Similarly, the *clec-41(tm6722)*;*clec-42(tm6526)* double mutant was as resistant as wildtype animals in one run and more resistant in another run (S5E Fig). Thus, there is a discrepancy between *clec-41;clec-42* double knock-out and knock-down phenotypes. This phenotypic difference between the *clec-41(tm6722)*;*clec-42(tm6526)* double knock-out mutant and simultaneous knock-down of *clec-41* and *clec-42* may be due to off-target effects of the RNAi treatment or to genetic compensation. We searched for potential off-targets of the RNAi clones but could not identify any targets in addition to *clec-41* and *clec-42*. Thus, it is possible that gene expression changes in other *clec* genes that mitigate the consequences of the *clec-41* and *clec-42* mutations and consequent compensation in the double mutant could be the reason for the observed differences. Genetic compensation or transcriptional adaptation in response to gene knock-out (but not gene knock-down) is a widespread phenomenon that has been observed in several model systems [88]. In *C*. *elegans*, genetic compensation was recently demonstrated for knock-down of *act-5* and *unc-89* [89]. If genetic compensation is indeed involved in the *clec-41(tm6722)*;*clec-42(tm6526)* double knock-out mutant needs to be determined in future. However, as the *clec* gene family is *C*. *elegans* 7^th^ largest gene family with 283 members, potential redundancy and complex interactions between different *clec* paralogs within the family may complicate the matter. Together, we conclude that analysis of knock-out mutants for genes of large gene families, such as the *clec* family, can have limited power for inferring gene functions, possibly because of gene compensation and/or redundancy. Therefore, we decided to further investigate the function of CLEC-4, CLEC-41, and CLEC-42 on the protein rather than the gene level.

### CLEC-41 and CLEC-42 bind a broad range of bacteria and exhibit antimicrobial activity *in vitro*

**Fig 6:**
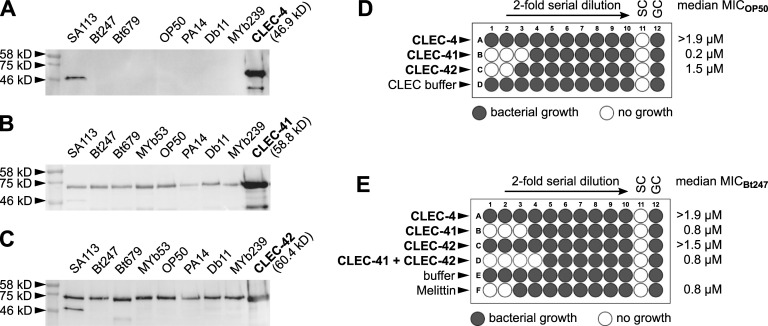
Recombinant CLEC-41 and CLEC-42 bind to bacteria and have antimicrobial activity *in vitro* against OP50 and Bt247. (A-C) 100 μl of Gram-positive (*S*. *aureus* SA113, *B*. *thuringiensis* Bt247, *B*. *thuringiensis MYBt18679*, and *Rhodococcus erythropolis* MYb53) and Gram-negative (*E*. *coli* OP50, *P*. *aeruginosa* PA14, *S*. *marcescens* Db11, and *S*. *rubidaea* MYb239) bacteria grown up to the mid-logarithmic phase were pelleted by centrifugation, washed and re-suspended in TBS buffer with CaCl_2_, and incubated with 6 μg recombinant protein with gentle rotation for 1 h at room temperature. Bacteria were then washed by centrifugation and subsequent resuspension of the pellet two times, eluted after a final centrifugation step with 100 μl of 2% SDS, subjected to SDS-PAGE and analyzed by Coomassie staining. Bound (A) CLEC-4, (B) CLEC-41, and (C) CLEC-42 proteins were detected through western blot with Mouse-anti-His mAb. Note that the band at approximately 46 kD in the SA113 lane is likely an artefact as it appears in each sample. (D, E) Recombinant CLEC-4, CLEC-41, and CLEC-42 were (D) 2-fold serially diluted in LB, and mixed with a bacterial suspension of OP50 in LB, or (E) mixed with a second CLEC protein or CLEC buffer, 2-fold serially diluted in CLEC buffer, and mixed with a bacterial suspension of Bt247 in LB. CLEC-4 did not inhibit bacterial growth, CLEC-41 and CLEC-42 visibly inhibited bacterial growth, individually and synergistically. The median MIC of (D) seven or (E) five experiments is shown, the buffer control refers to the proteins’ native buffer, Melittin served as antimicrobial positive control. SC = sterility control (only LB). GC = growth control (OP50 in LB). Also see S6 Fig and S3K Table.

Vertebrate CTLD proteins play important roles in pathogen recognition, acting as transmembrane PRRs or soluble PRRs and mediating intracellular signaling, but can also function as secreted antimicrobial proteins that kill bacteria [90]. Both functions imply direct binding to bacteria. As CLEC-4, CLEC-41 and CLEC-42 both have a signal peptide and are predicted to be secreted. To test the role of CLEC-4, CLEC-41, and CLEC-42 in bacterial recognition or elimination, we assessed binding of recombinant proteins (production of the individual proteins was outsourced to a specialized company; see materials and methods) to Gram-positive and Gram-negative bacteria. While the recombinant CLEC-4 protein did not bind any bacteria, CLEC-41 and CLEC-42 proteins bound to all tested bacteria (Fig 6A, 6B, and 6C). We next asked whether CLEC-4, CLEC-41, and CLEC-42 exhibit antimicrobial activity *in vitro*. While the CLEC-4 protein did not inhibit visible bacterial growth, CLEC-41 and CLEC-42 both exhibited antimicrobial activity against *E*. *coli* OP50, although only at higher concentrations (Fig 6D). Moreover, CLEC-41 inhibited visible growth of the pathogenic Bt247, but CLEC-42 did not (Figs 6E and S6B). Combining both proteins had a mild synergistic effect on Bt247 growth inhibition (Figs 6E and S6B), which is in line with the effect on survival upon joint silencing of *clec-41;clec-42* by RNAi (Fig 5A and 5B). In all cases, we were able to recover live bacteria from the wells without visible bacterial pellets, indicating that the CLEC proteins have bacteriostatic and not bactericidal activity. Interestingly, neither CLEC-41, nor CLEC-42 inhibited growth of Bt407 (S6A Fig), indicating specific interactions with different bacterial strains. While this is the first demonstration of antimicrobial function of *C*. *elegans* CLEC proteins, an *in vitro* bactericidal activity was previously described for several crustacean CTLD proteins, which also possess broad bacterial binding properties, for example Fc-hsL of the Chinese white shrimp *Fenneropenaeus chinensis* and EsLecA and EsLecG of the Chinese mitten crab *Eriocheir sinensis* that inhibit growth of Gram-positive as well as Gram-negative bacteria [91, 92].

In summary, CLEC-41 and CLEC-42 contribute to *C*. *elegans* resistance to Bt247 infection (Fig 5A and 5B), bind to a broad range of bacteria (Fig 6B and 6C) and exhibit weak antimicrobial activity against *E*. *coli* and pathogenic Bt247 *in vitro* (Fig 6D and 6E). Although it is possible that the native proteins may behave differently *in vivo*, our *in vitro* results for the recombinant proteins suggest that CLEC-41 and CLEC-42 are *bona fide* antimicrobial immune effector proteins.

## Conclusion

**Fig 7:**
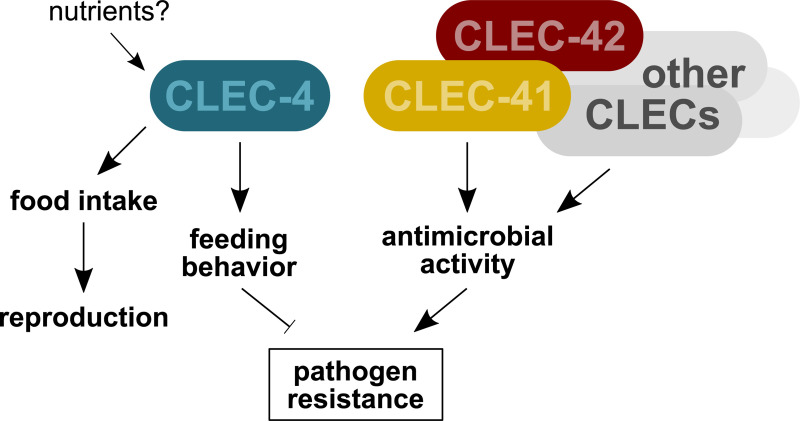
Model of CLEC-4, CLEC-41, and CLEC-42 function. *clec-4* is likely to regulate feeding behavior by controlling food intake and may be involved in sensing bacterial-derived or internal nutrients such as glucose or other glycans. *clec-4*-mutation-dependent decrease in bacterial load affects reproductive timing, yet also enhances pathogen resistance. CLEC-41 and CLEC-42 directly affect pathogen resistance through their antimicrobial activity against Bt247. Pathogen resistance might be further supported by other, closely related CLEC proteins, possibly in interaction and/or in a compensatory form with CLEC-41 and CLEC-42.

The exact functions of the extremely diversified *C*. *elegans clec* genes are largely unknown. Of the 283 *clec* gene family members only few have been studied at a functional genetic or protein level. Here, we explored the functions of CLEC-4, CLEC-41, and CLEC-42. We identified *clec-4* as a novel regulator of *C*. *elegans* feeding behavior and provide evidence of a link between *clec-4* function and nutrient sensing (Fig 7). Further, we show that *clec-41* and *clec-42* are required for resistance against Bt247 infection and demonstrate antimicrobial activity of CLEC-41 and CLEC-42 *in vitro* (Fig 7). Our work reveals a novel function of *C*. *elegans clec* genes in regulating feeding behavior, it defines a role for CLEC-41 and CLEC-42 as *bona fide* immune effector proteins, and thus, it extends the current knowledge on the functional diversification of this large gene family.

## Supporting information

S1 Fig *In vivo* expression of *clec-4* and *clec-43*. (A) Expression of *clec-4*p::GFP throughout the intestine in different *C*. *elegans* developmental stages. The co-injection marker *ttx-3*p::RFP is expressed in the AIY interneuron pair. All scale bars represent 20 μm. (B) *clec-43*p::GFP is exclusively expressed in the first intestinal ring (int1). The scale bar represents 20 μm.
(JPG)

S2 Fig Gene structure and genetic confirmation of *clec* knock-out mutants and RNAi-treated worms. (A-F) Gel electrophoresis pictures of RT-PCRs performed with either genomic DNA (gDNA) or copy DNA (cDNA) of the respective (A-E) knock-out mutant, the wildtype strain N2, or (F) RNAi-treated worms. NTC = no template control. (G) Gene structures of *clec* knock-out mutants used in the study with marked deletion alleles and RNAi clone insert target. The *ok2050* deletion is a large in-frame deletion of 1610 bp, removing one internal and the last exon. The *ya1* and *tm6526* deletions are in frame. The *tm6722* deletion is a frameshift mutation, leading to a premature stop codon. All deletions are expected to yield severely truncated proteins. However, no mutant mRNA transcripts could be detected by RT-PCR (A-E) and the deletions thus likely represent null alleles. The primer combinations are stated (A-F) on the bottom of the pictures and denoted (G) in the gene structure scheme. Also see S1A Table.
(JPG)

S3 Fig Resistance to Bt247 infection is the prevalent survival phenotype of *clec-4(ok2050)* and *clec-4(ya1)* mutants. (A, B) Here we present our survival data as heatmaps to facilitate the comparison of results between different knock-out mutants and highlight variation across technical and biological replicates. The heatmaps represent the difference of the area under the survival curve (AUC) of (A) *clec-4(ok2050)* and (B) *clec-4(ya1)* mutant worms versus the average of wildtype N2 worms from the same run (i.e., biological replicate). Purple and orange colors indicate the value of AUC difference. Purple indicates higher survival of mutant worms and orange indicates lower survival of mutant worms in comparison to wildtype N2 worms (see scale bar on the right side of (B)). Bars represent technical replicates. Asterisks show significant differences between mutant and N2. *p < 0.05, **p < 0.01, and ***p < 0.001, according to a generalized linear model (GLM) [57], where mutant worm strains were compared to wildtype. Run 1 (thick black border) in the heatmaps corresponds to results shown as survival curve in Fig 2C. (C) Difference in survival on serial dilutions of Bt247 and a dilution of 1:10 of the non-pathogenic Bt407 control 24 hpi between *clec-4(ya1)*, transgenic rescue strains for clec-4 MY1121 (*clec-4(ya1);unc-119(ed3);*yaEx111[*mtl-2*p::*clec-4;myo-2*p::RFP*;unc-119(+)*]) and MY1122 (*clec-4(ya1);unc-119(ed3);*yaEx112[*clec-4(+);myo-2*p::RFP*;unc-119(+)*]), and N2. Means ± standard deviation (SD) of n = 4 are shown, data are representative of two independent experiments. ***p < 0.001, according to a generalized linear model (GLM) [57]. Also see S2E, S4A and S4B Tables.
(JPG)

S4 Fig *clec-4* mutants show a decreased early offspring production. (A, B) Lifespan analysis of *clec-4(ok2050)* and *clec-4(ya1)* mutants and N2 wildtype animals under standard conditions. Repeated lifespan analyses led to inconclusive results despite of significant differences, n = 5, data are representative of three independent experiments (also see Fig 4C). **p < 0.01, according to Kaplan-Meier analysis [55] and log-rank test [56]. Horizontal ticks represent censored data (missing worms). (C, D) Brood size of *clec-4(ok2050)* and *clec-4(ya1)* mutants, and N2 wildtype animals (C) the first three days post L4 or (D) plotted across time. Shown are pooled data, n = 30 combined from three independent experiments. ***p < 0.001 as determined by Wilcoxon rank sum test, Bonferroni corrected. Also see Fig 4D, and S2F and S4C Tables.
(JPG)

S5 Fig Knock-down and knock-out of *clec-41* and *clec-42* alone and simultaneously have a variable effect on resistance to Bt247 infection. Difference in survival at 24 hpi between (A, B) *clec-41;clec-42(RNAi)* worms and empty vector control worms on (A) RNAi clone 1 (Ahringer library V-8P17) or (B) RNAi clone 2 (Ahringer library V-11P18), (C) single mutants *clec-41(tm6722)*, (D) *clec-42(tm6526)*, and (E) double mutants *clec-41(tm6722);clec-42(tm6526)* and wildtype N2 animals. Data represented in heatmaps and statistics as in S3 Fig. Asterisks show significant differences between mutant/RNAi worms and wildtype/RNAi control. *p < 0.05, **p < 0.01, and ***p < 0.001, according to a generalized linear model (GLM) [57]. (A, B) Run 1 (thick black border) corresponds to results shown as survival curve in Fig 5A and 5B. Also see S2G and S4D–S4H Tables.
(JPG)

S6 Fig Recombinant CLEC proteins do not have antimicrobial activity *in vitro* against Bt407. **(A**) Recombinant CLEC-4, CLEC-41, and CLEC-42 were mixed with a second CLEC protein or CLEC buffer, 2-fold serially diluted in CLEC buffer, and mixed with a bacterial suspension of Bt407 in LB. CLEC-4, CLEC-41, and CLEC-42 did not inhibit bacterial growth. The MIC of two independent experiments is shown, the buffer control refers to the proteins’ native buffer. SC = sterility control (only LB). GC = growth control (OP50 in LB). (B) Example of a MIC assay with Bt247 as shown in Fig 6. Also see S4I Table.
(JPG)

S1 Table Transgenic *clec* gene reporter and rescue strains, and primer sequences for generation of transgenic constructs by PCR fusion as well as for worm strain genotyping. (XLSX)

S2 Table Results of all statistical tests. (XLSX)

S3 Table Raw data for main Figs. (XLSX)

S4 Table Raw data for supporting Figs. (XLSX)

S5 Table Differential gene expression analysis of *clec-4(ok2050)* on non-pathogenic Bt407, including GO and WormExp EASE enrichment analyses. (XLSX)
