# Supplementary figures and images for: Effector and regulator: Diverse functions of C. elegans C-type lectin-like domain proteins
Source: PLoS Pathog. 2021 Apr 1;17(4):e1009454. doi: 10.1371/journal.ppat.1009454 (PMC8051790; doi:10.1371/journal.ppat.1009454)

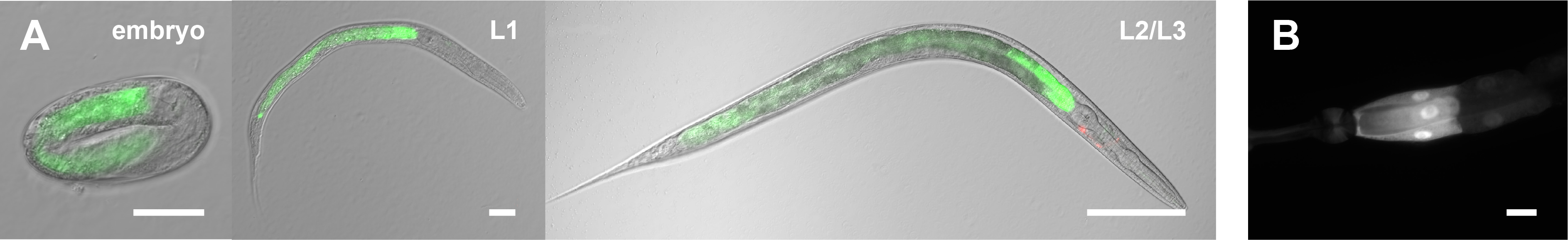

Supplement: S1 Fig — (A) Expression of clec-4p::GFP throughout the intestine in different C. elegans developmental stages. The co-injection marker ttx-3p::RFP is expressed in the AIY interneuron pair. All scale bars represent 20 μm. (B) clec-43p::GFP is exclusively expressed in the first intestinal ring (int1). The scale bar represents 20 μm. (JPG) [file ppat.1009454.s001.jpg]

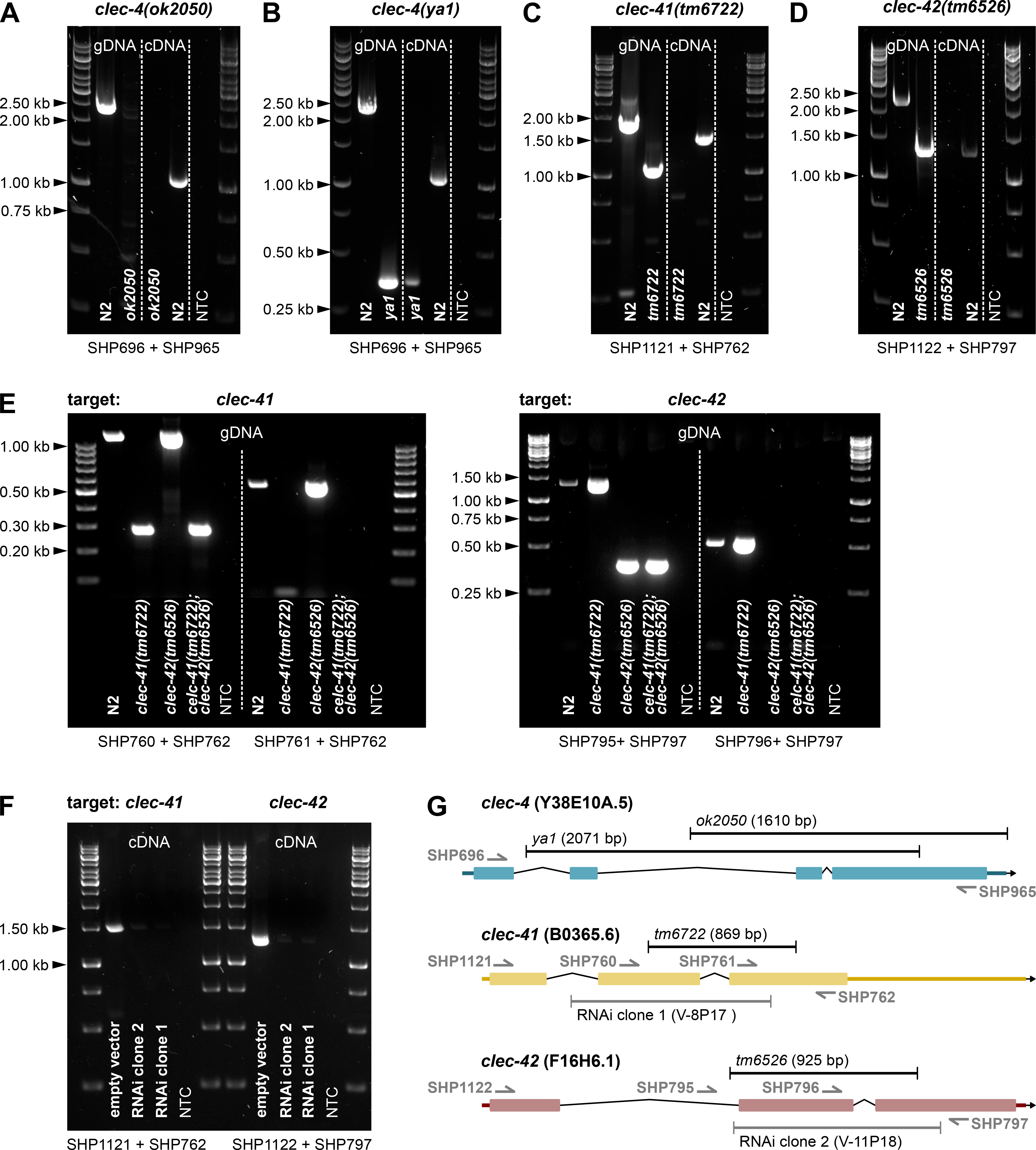

Supplement: S2 Fig — (A-F) Gel electrophoresis pictures of RT-PCRs performed with either genomic DNA (gDNA) or copy DNA (cDNA) of the respective (A-E) knock-out mutant, the wildtype strain N2, or (F) RNAi-treated worms. NTC = no template control. (G) Gene structures of clec knock-out mutants used in the study with marked deletion alleles and RNAi clone insert target. The ok2050 deletion is a large in-frame deletion of 1610 bp, removing one internal and the last exon. The ya1 and tm6526 deletions are in frame. The tm6722 deletion is a frameshift mutation, leading to a premature stop codon. All deletions are expected to yield severely truncated proteins. However, no mutant mRNA transcripts could be detected by RT-PCR (A-E) and the deletions thus likely represent null alleles. The primer combinations are stated (A-F) on the bottom of the pictures and denoted (G) in the gene structure scheme. Also see S1A Table. (JPG) [file ppat.1009454.s002.jpg]

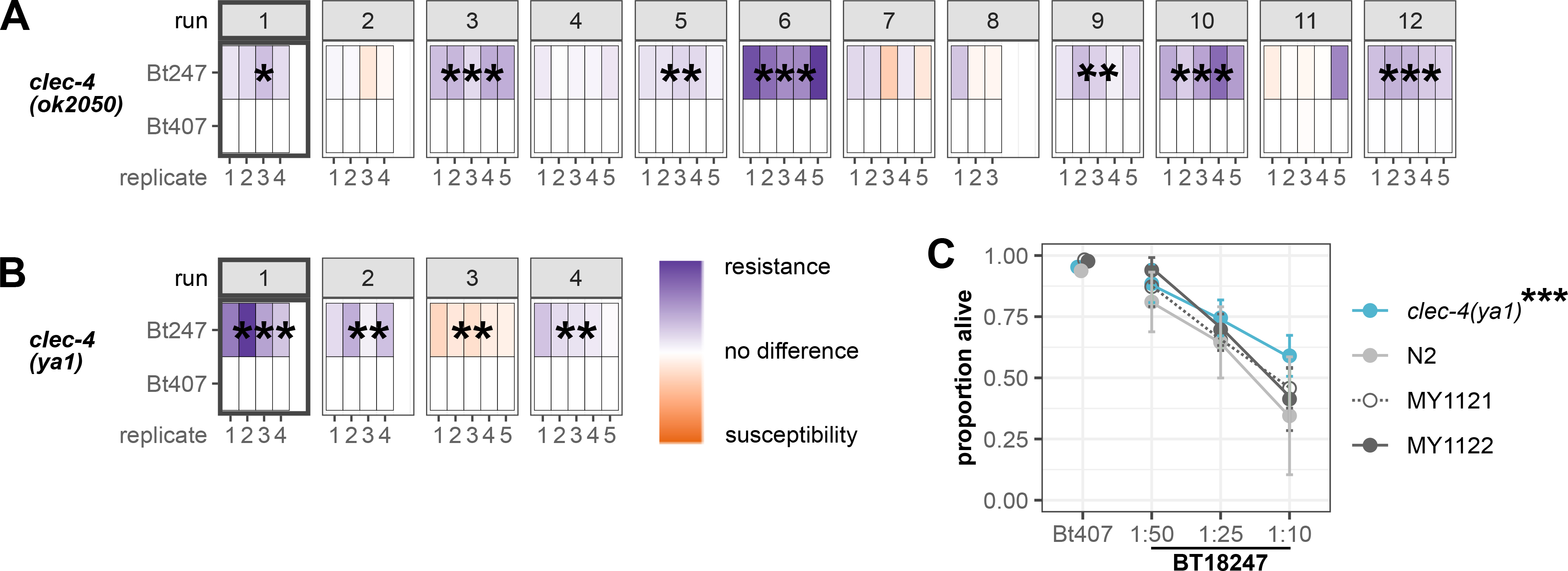

Supplement: S3 Fig — (A, B) Here we present our survival data as heatmaps to facilitate the comparison of results between different knock-out mutants and highlight variation across technical and biological replicates. The heatmaps represent the difference of the area under the survival curve (AUC) of (A) clec-4(ok2050) and (B) clec-4(ya1) mutant worms versus the average of wildtype N2 worms from the same run (i.e., biological replicate). Purple and orange colors indicate the value of AUC difference. Purple indicates higher survival of mutant worms and orange indicates lower survival of mutant worms in comparison to wildtype N2 worms (see scale bar on the right side of (B)). Bars represent technical replicates. Asterisks show significant differences between mutant and N2. *p < 0.05, **p < 0.01, and ***p < 0.001, according to a generalized linear model (GLM) [57], where mutant worm strains were compared to wildtype. Run 1 (thick black border) in the heatmaps corresponds to results shown as survival curve in Fig 2C. (C) Difference in survival on serial dilutions of Bt247 and a dilution of 1:10 of the non-pathogenic Bt407 control 24 hpi between clec-4(ya1), transgenic rescue strains for clec-4 MY1121 (clec-4(ya1);unc-119(ed3);yaEx111[mtl-2p::clec-4;myo-2p::RFP;unc-119(+)]) and MY1122 (clec-4(ya1);unc-119(ed3);yaEx112[clec-4(+);myo-2p::RFP;unc-119(+)]), and N2. Means ± standard deviation (SD) of n = 4 are shown, data are representative of two independent experiments. ***p < 0.001, according to a generalized linear model (GLM) [57]. Also see S2E, S4A and S4B Tables. (JPG) [file ppat.1009454.s003.jpg]

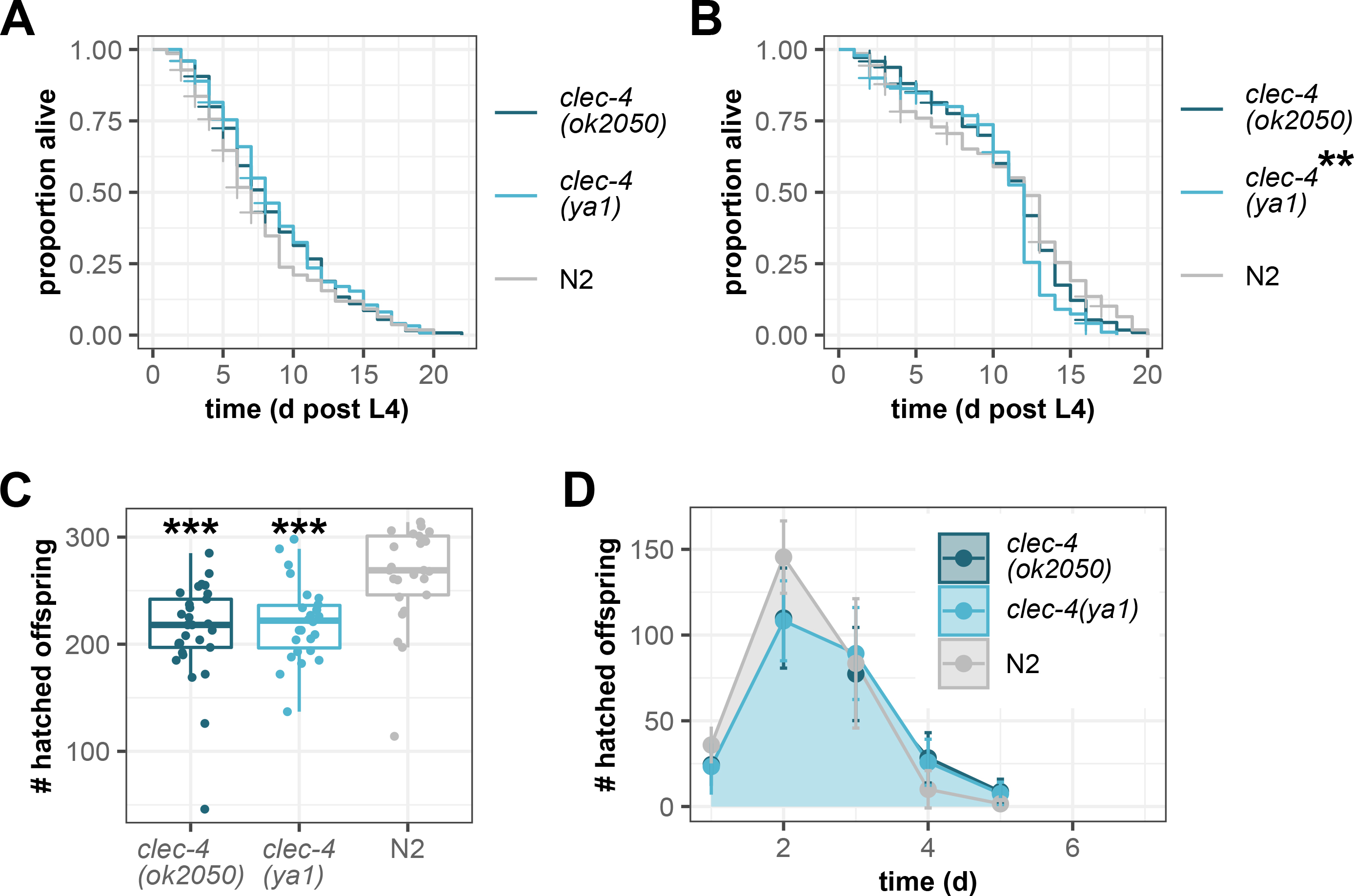

Supplement: S4 Fig — (A, B) Lifespan analysis of clec-4(ok2050) and clec-4(ya1) mutants and N2 wildtype animals under standard conditions. Repeated lifespan analyses led to inconclusive results despite of significant differences, n = 5, data are representative of three independent experiments (also see Fig 4C). **p < 0.01, according to Kaplan-Meier analysis [55] and log-rank test [56]. Horizontal ticks represent censored data (missing worms). (C, D) Brood size of clec-4(ok2050) and clec-4(ya1) mutants, and N2 wildtype animals (C) the first three days post L4 or (D) plotted across time. Shown are pooled data, n = 30 combined from three independent experiments. ***p < 0.001 as determined by Wilcoxon rank sum test, Bonferroni corrected. Also see Fig 4D, and S2F and S4C Tables. (JPG) [file ppat.1009454.s004.jpg]

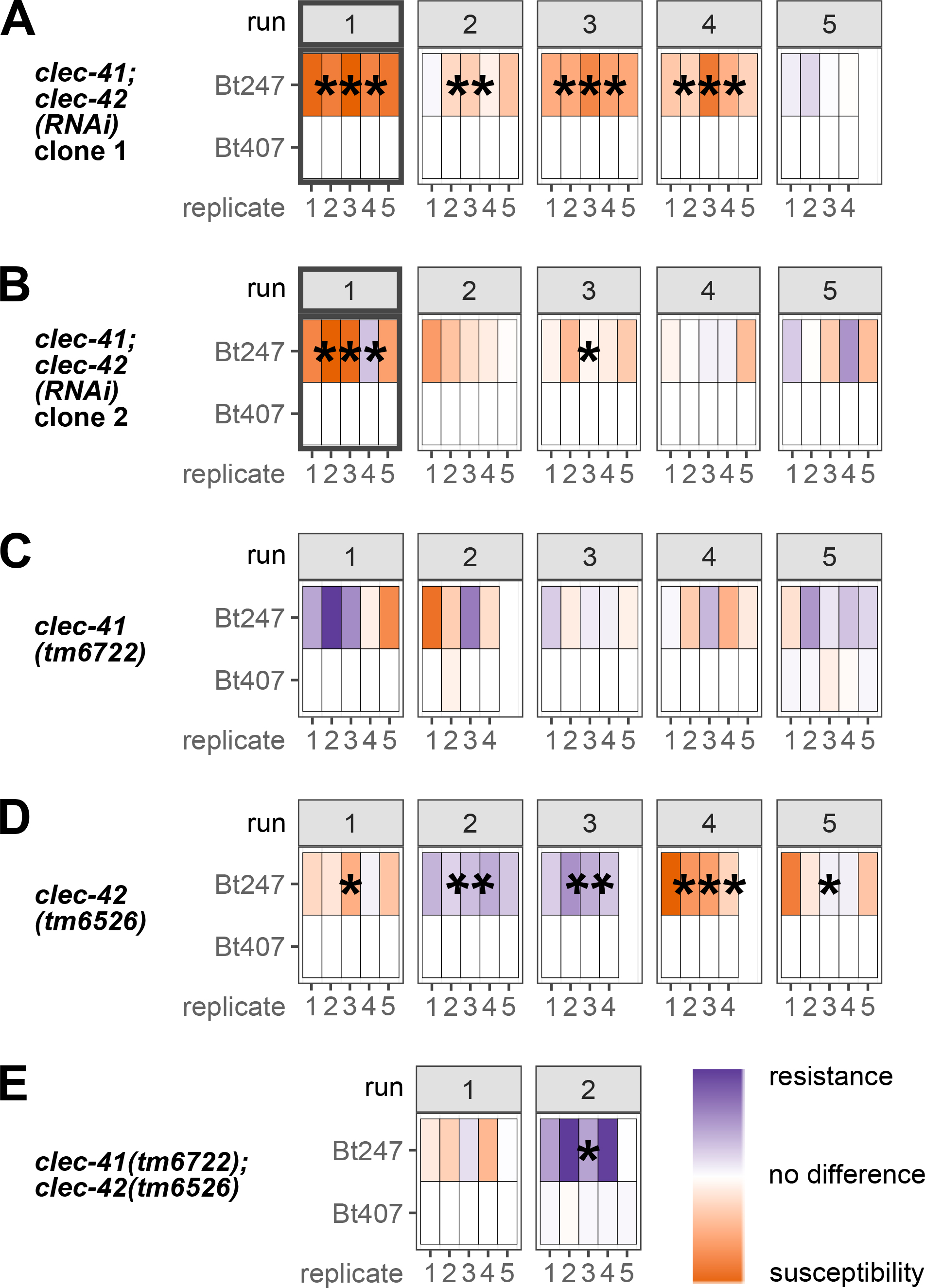

Supplement: S5 Fig — Difference in survival at 24 hpi between (A, B) clec-41;clec-42(RNAi) worms and empty vector control worms on (A) RNAi clone 1 (Ahringer library V-8P17) or (B) RNAi clone 2 (Ahringer library V-11P18), (C) single mutants clec-41(tm6722), (D) clec-42(tm6526), and (E) double mutants clec-41(tm6722);clec-42(tm6526) and wildtype N2 animals. Data represented in heatmaps and statistics as in S3 Fig. Asterisks show significant differences between mutant/RNAi worms and wildtype/RNAi control. *p < 0.05, **p < 0.01, and ***p < 0.001, according to a generalized linear model (GLM) [57]. (A, B) Run 1 (thick black border) corresponds to results shown as survival curve in Fig 5A and 5B. Also see S2G and S4D–S4H Tables. (JPG) [file ppat.1009454.s005.jpg]

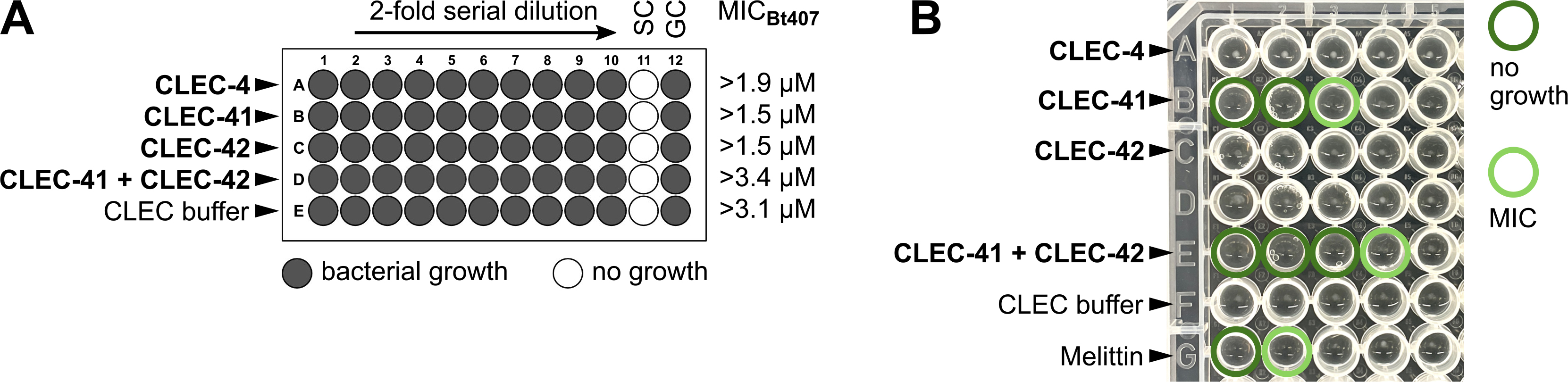

Supplement: S6 Fig — (A) Recombinant CLEC-4, CLEC-41, and CLEC-42 were mixed with a second CLEC protein or CLEC buffer, 2-fold serially diluted in CLEC buffer, and mixed with a bacterial suspension of Bt407 in LB. CLEC-4, CLEC-41, and CLEC-42 did not inhibit bacterial growth. The MIC of two independent experiments is shown, the buffer control refers to the proteins’ native buffer. SC = sterility control (only LB). GC = growth control (OP50 in LB). (B) Example of a MIC assay with Bt247 as shown in Fig 6. Also see S4I Table. (JPG) [file ppat.1009454.s006.jpg]
